# Effects of Temperature and Host Concentration on the Supramolecular Enantiodifferentiating [4 + 4] Photodimerization of 2-Anthracenecarboxylate through Triplet-Triplet Annihilation Catalyzed by Pt-Modified Cyclodextrins

**DOI:** 10.3390/molecules24081502

**Published:** 2019-04-17

**Authors:** Ming Rao, Wanhua Wu, Cheng Yang

**Affiliations:** Key Laboratory of Green Chemistry & Technology, College of Chemistry, and Healthy Food Evaluation Research Center, Sichuan University, 29 Wangjiang Road, Chengdu 610064, China; raomingrm@163.com

**Keywords:** photochirogenesis, supuramolecular chemistry, chiral photochemistry, catalysis, TTA, host-guest, 2-anthracenecarboxylate

## Abstract

Visible-light-driven photocatalytic supramolecular enantiodifferentiating dimerization of 2-anthracenecarboxylic acid (AC) through triplet-triplet annihilation (TTA), mediated by the Schiff base Pt(II) complex (**Pt-1**, **Pt-2**, and **Pt-3**) was studied. The host concentration and the temperature effects on the stereoselectivity were comprehensively investigated. Increasing the concentration of sensitizers/hosts significantly enhanced the conversion of the photoreaction but led to reduced enantioselectivities of the chiral photodimers **2** and **3** when the photoreaction was triggered by a 532 nm laser, which was in contrast with the results obtained by direct irradiation of AC with a 365 nm light-emitting diode (LED) lamp, due to the aggregation of the sensitizer/host in water. The cyclization of AC through triplet-triplet annihilation displayed significant temperature dependency when **Pt-3** was employed as the sensitizer/host. Increasing the temperature from 0 °C to 30 °C with 5% equiv. of **Pt-3** led to a great increase of the ee of **2** from 2.1% to 31.6%. However, hardly any temperature dependency was observed when the photodimerization was mediated by other sensitizers and/or hosts, or the photoreaction was triggered directly with a 365 nm LED lamp.

## 1. Introduction

Asymmetric photochemical reaction is particularly intriguing due to its unique ability to gain chiral polycyclic, highly constrained and/or thermally unstable organic compounds which are difficult to synthesize by traditional thermochemical or enzymic reactions [1]. However, the short-lived, highly reactive and weakly interactive properties of the excited photosubstrates make it extremely challenging to manipulate the enantioselectivity of excited state reactions. Supramolecular photochirogenesis provides a promising solution to such problems, as “confined space” for the prochiral substrate(s) was created before photoexcitation through the much longer and more intimate supramolecular interactions at both ground and excited states. A variety of chiral hosts, such as cyclodextrin derivatives [2,3,4,5,6,7,8,9,10,11,12,13,14,15], chiral hydrogen-bonding templates [16,17,18,19], chiral macrocyclic molecules [20,21] and biological macromolecules [22,23], have been developed and utilized as chiral sources for efficient photochirogenic control. However, an excess amount of chiral host is demanded in most supramolecular photochirogenesis for the purpose of inhibiting the undesired racemic photoproduct resulting from the photoreaction of the uncomplexed photosubstrates in the bulk solution [24]. Catalytic supramolecular photochirogenesis is more desirable considering the not easy accessibility and atom economy of the chiral hosts, which is however much more difficult to realize [25,26]. Several endeavors have been made to achieve catalytic enantiodifferentiating photoreaction, and among them photosensitization is the most frequently applied strategy [27]. Supramolecular photosensitization has been realized through the much more efficient in situ electron and/or energy transfer from the hydrophobic sensitizer covalently grafted on the host to the substrates embedded in the host cavity. Moreover, photocatalysis also has been realized by sophisticated wavelength control to provide the possibility to exclusively excite the complexed substrate and avoid the background reaction [28].

We have recently reported the first triplet-triplet annihilation (TTA)-based catalytic photochirogenesis exemplified with the enantiodifferentiating photodimerization of 2-anthracenecarboxylic acid (AC) sensitized by Schiff base Pt(II) complex-grafted γ-cyclodextrins (CDs) [29], which combined the advantages of the supramolecular photosensitization and wavelength control strategies. TTA upconversion is a well-developed technology to spectrally blue-shifting the wavelength of the excitation photons [30,31], which have been comprehensively applied in various fields, such as solar cells [32,33], photocatalysis [34] and bioimaging [35]. TTA-based catalytic photochirogenesis was realized through the following 4 steps: (i) the photosubstrate and the sensitizer-grafted hosts form a host-guest complex; (ii) excitation of the photosensitizers by the low-power continuous-wave laser and the triplet-states of the photosensitizers was populated through intersystem crossing (ISC); (iii) the triplet energy of the sensitizer was in situ transferred to the complexed acceptor and (iv) two triplet acceptors generate a singlet acceptor through TTA while the other deactivates to the ground state, and the highly reactive singlet acceptor will dimerize with the deactivated acceptor to give the photodimers. A nice trick for this strategy is that the triplet sensitizer was exclusively photoexcited, and the triplet energy transfer from the sensitizer to the substrate in the cavity of the hosts will be much more effective than to the unbound substrate [36,37], and, therefore, good enantioselectivity has been achieved with photocatalyst as low as 0.5% equivalent.

**Scheme 1:**
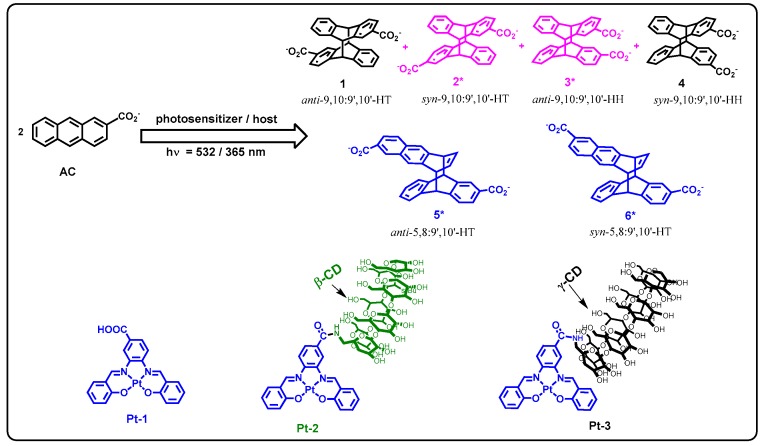
Enantiodifferentiating [4 + 4] photocyclodimerization of 2-anthracenecarboxylate (AC) mediated by photosensitizers **Pt-1**, **Pt-2**, and **Pt-3**.

Supramolecular enantiodifferentiating [4 + 4] photocyclodimerization of AC has been established as the benchmark reaction for photochirogenesis [24,38,39,40,41]. This photoreaction will afford classical cyclodimers **1**–**4**, among which *syn*-9,10:9′10′-*head-to-tail* (*syn*-9,10:9′10′-HT) dimer **2** and *anti*-9,10:9′10′-*head-to-head* (*anti*-9,10:9′10′-HH) dimer **3** are chiral, and the absolute configuration of these photoreaction products were determined on the basis of theoretical and experimental comparsion of circular dichroism spectra [42]. It is also reported that AC can form 2:2 high order complex with β-CD to obtain irregular cyclodimers *anti*-5,8:9′10′-*head-to-tail* (*anti*-5,8:9′10′-HT) dimer **5** and *syn*-5,8,9′10′-*head-to-tail* (*syn*-5,8:9′10′-HT) dimer **6** (Scheme 1) [43,44,45]. Herein, a new sensitizing chiral host **Pt-2** was synthesized by grafting β-CD to the photosensitizer **Pt-1** (Scheme S1) to realize photocatalytic supramolecular enantiodifferentiating dimerization of AC triggered by low-power visible light and better TTA efficiency is expected, moreover, the temperature effects on the photoreaction mediated by the chiral sensitizers (**Pt-2** and **Pt-3**) were systematically investigated to gain further insight into the mechanisms of the supramolecular catalytic photochirogenesis through TTA.

## 2. Results and Discussion

### 2.1. Enantiodifferentiating Photocyclodimerization of 2-Anthracenecarboxylate (AC) Mediated by **Pt-2**

#### 2.1.1. Characterization of **Pt-2**

**Figure 1:**
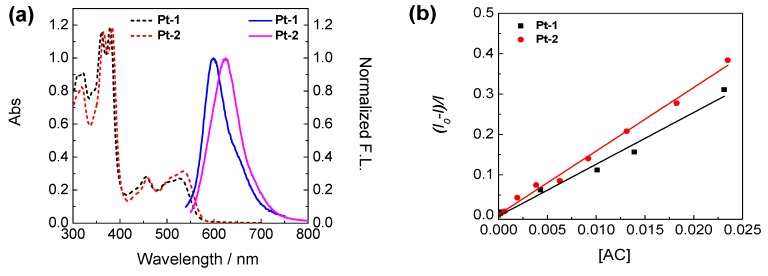
(**a**) Ultraviolet-visible (UV-vis) absorption spectra (dotted line) and normalized emission spectra (solid line) of **Pt-1** and **Pt-2,** *C* = 5 × 10^−5^ M, 20 °C. (**b**) Stern-Volmer Plots of (*I*_0_ − *I*)/*I* versus [AC] in deaerated water, *C*_[**Pt-1**, **Pt-2**]_ = 5 × 10^−5^ M, *C*_[AC]_ = 0 − 2.31 × 10^−5^ M, 20 °C.

Schiff base Pt(II) complex photosensitizer grafted β-CD (**Pt-2**) was synthesized by reacting **Pt-1** with 6^A^-amino-6^A^-deoxy-β-CD and was used as a photosensitive chiral host to trigger a visible-light-driven photocyclodimerization of AC. **Pt-1** was also investigated as a reference sensitizer for the comparison purpose. **Pt-2** showed moderate visible absorption and intensive red phosphorescence in deaerated aqueous solution (Figure 1a), and the maximum absorption and phosphorescence were slightly red-shifted relative to **Pt-1**. In addition, **Pt-2** shows thermally induced Stokes shifts (Δ*E_S_*) and structured emission spectra (Figure S7), indicating a metal-to-ligand charge transfer (MLCT) feature of the triplet excited state. The triplet state lifetimes slightly longer probably due to the partial self-inclusion of Schiff-base unit by β-CD (Table S1) [46]. The triplet energy of **Pt-2**, estimated from the phosphorescent emission spectra, was 2.03 eV, which is higher than that of AC (1.82 eV), guaranteeing an efficient triplet-triplet energy transfer to AC. To investigate the energy transfer efficiency, phosphorescence quenching titration of photosensitizers **Pt-1** and **Pt-2** by acceptor AC were performed (Figure S8). The Stern–Volmer analysis of the emission quenching data showed an apparent linear relationship (Figure 1b), from which the Stern–Volmer constants *K_SV_* were derived. **Pt-2** showed a *K_SV_* value of 1.57 × 10^4^ M^−1^, being slightly larger than that of **Pt-1** (1.28 × 10^4^ M^−1^), for which the host-guest interaction between AC and **Pt-2**, as well as the slightly longer triplet lifetime of **Pt-2** (τ_Pt-1_ = 2.0 μs, τ_Pt-2_ = 3.1 μs) should be responsible [47]. 

#### 2.1.2. Effects of **Pt-2**/AC Ratio 

Photolyses of AC in the presence of **Pt-1** and **Pt-2** were carried out by using a 532 nm diode pumped solid state laser or a 365 nm LED lamp in pH 9.0 borate buffer solution. The photoreaction results were shown in Table 1. Photosensitization of 0.2 mM AC with 0.05 mM **Pt-2** for 2 h with 532 nm laser led to 80% conversion, which is higher than that obtained with **Pt-1** (53%), and could be ascribed to the improved triplet-triplet energy transfer (TTET) efficiency by the host–guest complexation between β-CD and AC [48]. This host–guest interaction was further verified by NMR studies, as all of the ^1^H-NMR signals for aromatic protons of AC were shifted upfield upon the complexation with **Pt-2** (Figure S5). In order to determine the inclusion rate between **Pt-2** and AC, the ultraviolet-visible (UV-vis) spectral Job plots for the solutions of AC and **Pt-2** at a fixed total concentration of 0.2 mM showed that the maximum UV−vis changes at 0.5 molar fraction, demonstrating a 1:1 complexation stoichiometry (Figure S10a). Unfortunately, due to the poor solubility of **Pt-2** in water, we failed to get the binding constants of **Pt-2** and AC, either by UV-vis or isothermal titration calorimeter (ITC) titration. We have demonstrated that AC forms 2:2 complex with β-CD, in which two AC molecules slipped stacked in the capsule formed by two β-CD cavities [49], which led to the slipped photodimers **5** and **6** when the complex was directly photolyzed by using a 365 nm LED. Interestingly, photodimerizing AC (0.2 mM) in the presence of 0.1 mM **Pt-2** only afforded the normal photodimers **1**–**4**, completely no slipped photodimers **5** or **6** were observed, in all photolyses either using the 365 nm LED lamp or 532 nm laser as the light source. This result demonstrates that **Pt-2** is more difficult to form 2:2 complex with AC than native β-CD.

On the other hand, photodimerize of AC (0.2 mM) in the presence of catalytic amount of **Pt-2** (0.01 mM) by using the 532 nm laser led to HT dimer **2** in 2.2% ee and HH dimer **3** in −2.0% ee. This result demonstrated a chirality transfer from **Pt-2** to AC dimers and the fact that the AC pair included in the capsule of two **Pt-2** are not slip arranged. The circular dichroism spectrum of **Pt-2** showed a negative signal at the MLCT transition (500−580 nm) which is different from **Pt-3** (Figure S8), according to the “sector rule” proposed by Kajitar [50], we conclude that the sensitizer’s square-coordinated plane reclines on the primary rim of β-CD and partially inserting into β-CD cavity and this probably prohibit the formation of cyclodimers **5** and **6**.

The host concentration effect on the photoreaction of AC sensitized by **Pt-2** was further investigated. When irradiating with a 532 nm laser and increase the concentration of **Pt-2** from 0.001 mM to 0.1 mM significantly increased the conversion from 1% to 97%, suggesting the acceleration effect of **Pt-2** towards the cyclization reaction of AC, the enantioselectivity of the photoreaction was slightly changed with the increasing of the concentration of **Pt-2,** with the ee value of dimer **3** increased from −0.2% to −3.5%, and dimer **2** changed from 1.6% to 1.8%. When the light source changed to a LED lamp at 365 nm, however, the ee values of dimer **3** increased with the increment of the concentration of the sensitizer/host **Pt-2**, and the change of dimer **2** was inconsistent with that of the 532 nm laser (Table 1). Considering the relatively low ee values, every experiment was carried out independently for 3 times. The ee values from the three times experiments agree very well and the average values are listed in Table 1. The altered chiral environment due to the aggregation of **Pt-2** with increased concentration should be partially responsible for the changed optical outcomes. The aggregation of **Pt-2** in aqueous solution was supported by the following facts: (i) the ^1^H nuclear magnetic resonance (NMR) signals of the aromatic protons of **Pt-2** showed clear peaks in DMSO-*d*_6_, when increasing the amount of D_2_O, the signals shifted upfield and became broad, and in D_2_O there is no signals observed (Figure S4), (ii) UV-vis absorption maxima showed a bathochromic shift of 11 nm upon increasing the concentration of **Pt-2** from 1 μM to 20 μM and then a modest bathochromic shift of only 2 nm upon further increasing the concentration to 500 μM (Figure S6b), demonstrating that **Pt-2** begins to form aggregation at concentration as low as 20 μM. On the other hand, as the optical outcomes of both **2** and **3** demonstrated completely different variation trend vs. the concentration of **Pt-2** when irradiated by a 532 nm laser and a 365 nm LED, we conclude that maybe photocyclodimerization of AC sensitized by **Pt-2** through TTA underwent different mechanisms comparing with that by direct irradiation with 365 nm LED.

#### 2.1.3. Effects of Temperature

In order to gain further insight into the mechanisms of the catalytic photodimerization of AC through triplet-triplet annihilation, we studied the influence of temperature on this photoreaction. The photocyclodimerization of AC was performed over a temperature range of 0–30 °C in the presence of 0.01 mM or 0.1 mM **Pt-2**, by irradiation with 532 nm laser or 365 nm LED lamp and the results were listed in Table 2. Lowering the temperature did not significantly change the product distribution but enhanced the enantioselectivities of the dimers **2** and **3**, regardless by sensitization process through TTA or by direct irradiation of AC with a 365 nm LED lamp. For instance, the ee value of dimer **2** increased from −0.7% to −7.9% and dimer **3** was increased from 1.2% to 5.9% by direct irradiation of 0.2 mM AC with 0.1 mM **Pt-2** with 365 nm LED lamp when the temperature decreased from 30 °C to 0 °C, while for the sensitization process, the ee value of dimer **2** was increased from 0.3% to 5.0%. It is noteworthy that photocyclodimerization of AC through TTA sensitization is less sensitive to the host concentration change, as photoirradiation of 0.01 mM **Pt-2** and 0.2 mM AC by a 532 nm laser afforded **2** in 3.7% ee, while direct irradiation of AC only led to 1.6% ee under the same condition, which indicates the catalytic characteristics for the cyclization of AC through TTA.

**Figure 2:**
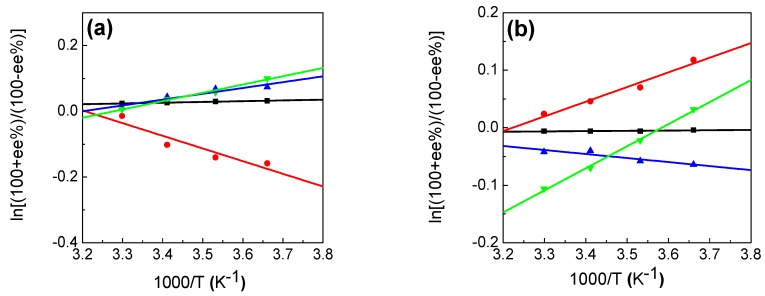
Representative temperature-dependence (differential Eyring) plots (**a**) of the ee of **2**, (**b**) of the ee of **3** obtained in the photoreaction of AC irradiated with 365 nm LED and 0.01 mM **Pt-2** (■) or 0.1 mM **Pt-2** (**●**), with 532 nm laser and 0.01 mM **Pt-2** (**▲**) or 0.1 mM **Pt-2** (**▼**) in 25 mM borate buffer solution.

To analyze the temperature effect quantitatively, we plotted the natural logarithm of the relative enantiomer ratios of dimer **2** and **3**, as a function of the reciprocal temperature, as illustrated in Figure 2, all of the data obtained with different chiral host concentrations and excitation wavelengths in borate buffer solution over a temperature range of 0–30 °C, gave excellent straight lines. According to the Eyring equation, the differential activation enthalpy (ΔΔ*H*^‡^) and entropy changes (ΔΔ*S*^‡^) were calculated from the intercept and slope of the plots and were listed in Table 3 [29].

In general, the residence time of AC in the host–guest complex is much longer than the lifetime of a singlet excited AC molecule (16 ns), and AC pairs is not possible to exchange their relative arrangement during the short excited lifetime. The product ee is primarily decided by the stability difference of the two precursory diastereomeric host-guest complexes. For the photoreaction through TTA upconversion, however, as the lifetime of the reactive singlet excited AC arising from the triplet-triplet annilation upconverison will fall in the μs–ms range, which is much longer than that of the singlet state formed by direct irradiation and is comparable with the dissociation of AC from the complexes (10^−3^~10^−6^ s depending on the association constants). Therefore, the kinetics of AC entering into the cavity of β-cyclodextrin may play a role in influencing the reaction stereoselectivity. However, due to the poor solubility of **Pt-2** in water, we are not able to obtain the exact binding constants between **Pt-2** and AC to get the accurate dissociation time. Hence, we turned our research focus on the temperature dependence of the photocyclodimerization of AC sensitized by **Pt-3**, which give much better water solubility and the binding constants of AC, to get deep insight into the mechanisms of the catalytic photoreaction through TTA.

### 2.2. Temperature Dependence of the Photocyclodimerization of AC Mediated by **Pt-3**

**Pt-3** sensitized TTA dimerization of AC have been reported by us to afford higher enantioselectivity for HT dimer **2** in the presence of catalytic amounts of chiral hosts when irradiated by a 532 nm solid laser [29]. Indeed, photocyclodimerization of AC in the presence of 1 mM **Pt-3** gave HT dimer **2** in only 16.9% ee, while reducing the concentration of **Pt-3** to 0.01 mM, which is 5% equiv. of AC, the ee of **2** can be enhanced to 31.6% ee at 30 °C. We ascribed this host concentration effect to the aggregation of **Pt-3** at high concentrations, which was further confirmed by the photoreaction of AC mediated by **Pt-2** in the present study. 

**Figure 3:**
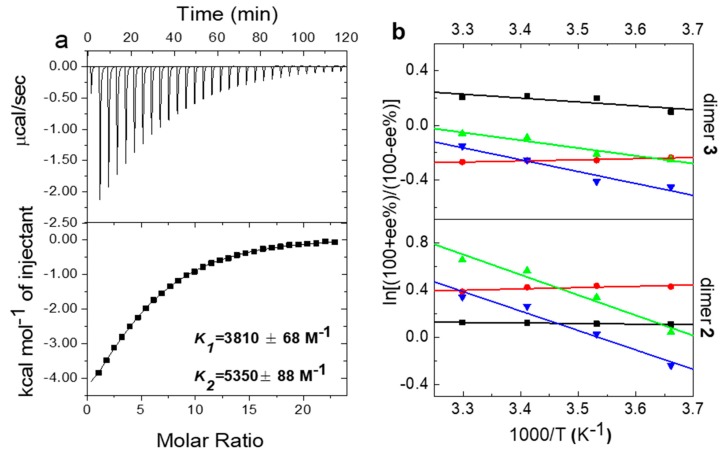
(**a**) Isothermal titration calorimeter (ITC) data for **Pt-3** (2 mM) and AC (0.2 mM) in 25 mM borate buffer (pH 9.0) at 0 °C. The top is the raw data for power versus time, and the bottom is the integrated enthalpy values versus the molar ratio of **Pt-3**: AC = 1:2, and obtained *K*_1_ as 3810 M^−1^, *K*_2_ as 5350 M^−1^. (**b**) Representative temperature-dependence (differential Eyring) plots obtained in the photoreaction of AC irradiated with 365 nm LED and 0.01 mM **Pt-3** (■) or 1 mM **Pt-3** (**●**), with 532 nm laser and 0.01 mM **Pt-3** (**▲**) or 1 mM **Pt-3** (**▼**). The top is the ee of **2**, and the bottom is the ee of **3**.

Before we investigated the temperature-dependence behaviors of the enantiodifferentiating photodimerization of AC catalyzed by **Pt-3**, we firstly examined the binding behavior between **Pt-3** and AC. The inclusion rate between **Pt-3** and AC was determined by the Job’s plot, based on the continuous variation method by holding AC and **Pt-3** at a fix total concentration of 0.2 mM, the maximum UV−vis changes were at 0.67 molar fraction, demonstrated a 1:2 complexation stoichiometry (Figure S10b). The binding constants between **Pt-3** and AC at different temperatures of 0 °C and 25 °C was determined with an ITC (Figure 3a). **Pt-3** binds with AC stepwisely to give 1:1 (*K*_1_) and 1:2 (*K*_2_) association constants as 3810 M^−1^ and 5350 M^−1^ at 0 °C, respectively. While at 25 °C, *K*_1_ and *K*_2_ were determined as 10,400 M^−1^ and 719 M^−1^, respectively [43]. The overall association constant *K_1_K_2_* was determined as 2.04 × 10^7^ M^−2^ at 0 °C and 7.48 × 10^6^ M^−2^ at 25 °C (Table S2), which were comparable to that of native γ-CD (4.14 × 10^7^ M^−2^ at 5 °C and 6.20 × 10^6^ M^−2^ at 25 °C) [43], indicating that **Pt-3** remains strongly binding toward **AC**. Intrestingly, *K*_2_ at high temperature is much smaller than that at lower temperature, demonstrating that the residence time of AC in the 1:2 host-guest complex is much shorter at high temperatures, from which, we can legitimately infer that, the thermodynamic enantioselectivity derived from the stability difference of the prochiral complexes formed by enantioface-selective inclusion of the second AC molecule into a 1:1 **Pt-3**-AC complex will become less predominant. However, the enantioselectivity come from the kinetics of AC entering into the cavity of CD become more and more non-negligible by increasing the temperature; therefore, the selectivity of the enantiodifferentiating photodimerization of AC though TTA should display different temperature-dependent behaviors comparing with the photoreaction triggered with a 365 nm LED lamp.

The photocyclodimerization of AC mediated by **Pt-3** were carried out over the temperature ranges from 0–30 °C in borate buffer solution (pH 9.0) and the photoreaction results are listed in Table 4. Interestingly, in the photoreaction mediated by 0.01 mM **Pt-3** in 25 mM borate buffer solution (pH 9.0), increasing the reaction temperature from 0 °C to 30 °C, led to an increase of the ee value of HT dimer **2** from 2.1% to 31.6%, while the ee of HH dimer **3** decreased from −12.4% to −3.0%. Particularly, when employing 1 mM **Pt-3** as the sensitizer/host, lowering temperature from 30 to 0 °C afforded an antipodal product for HT dimer **2**. However, when the photoreaction was triggered by a 365 nm LED lamp, the outcomes of both dimers **2** and **3** showed no apparent temperature dependency at the temperature range of 0–30 °C (Table 5).

The apparently different temperature-dependent behaviors for **Pt-3** when irradiation with 532 nm laser comparing with the direct irradiation of AC with 365 nm LED lamp, should be ascribed to the different mechanisms of the photoreaction through TTA. For direct irradiation of AC, the enantioselectivity of the chiral product **2** and **3** derive from the thermodynamic stability of the prochiral 1:2 complex of γ-CD and AC, while for TTA-UC triggered photoreaction, the kinetics of AC entering into the cavity of γ-CD also played vital roles as TTA-UC comprise several inter-molecular energy transfer processes. Considering that the variation trend of the ee versus temperature is opposite when changing the exciting wavelength from 365 nm to 532 nm, we conclude that the kinetic process gave antipodal product to the thermodynamic control product, and kinetic control products is dominant for visible light-driven photoreaction mediated by **Pt-3**. When the temperature increases, molecular diffusion rate become higher, the kinetic control products become more dominant to give enhanced optical yields of **2** and **3.**

To more quantitively analyze the temperature-behavior of the chiral product **2** and **3**, the natural logarithm of the relative enantiomer ratios of the chiral dimers, obtained with the irradiation of 365 nm LED lamp and 532 nm laser mediated by different concentrations of **Pt-3**, were plotted against the reciprocal temperature (Figure 3b), all the fittings showed good linear relationship and from the slope and intercept of the fittings, differential activation enthalpy (ΔΔ*H*^‡^) and entropy changes (ΔΔ*S*^‡^) were calculated, the results were listed in Table 5. Interestingly, the photoreaction triggered by a 532 nm laser showed obviously large ΔΔ*H*^‡^ and ΔΔ*S*^‡^ value than that trigged by 365 nm irradiation. The 365 nm irradiation will produce a singlet excited AC, which has a short lifetime of 16 ns in aqueous solution. No rearrangement of AC pairs in the CD cavity are possible during this short time, and the differential thermodynamic parameters reflect more the difference between diastereomeric complexes at the ground state. For the 532 nm-induced photodimerization, the triplet excited AC has much longer lifetime, and it is possible to establish a new equilibrium at the excited state. The differential entropy and enthalpy values for 532 nm irradiation are mainly the activation differential thermodynamic parameters for the formation of new equilibrium at the excited state. The much larger differential entropy and enthalpy values under 532 nm irradiation indicated that activated state for the formation of triplet-excited AC pairs are greatly different from each other. 

**Figure 4:**
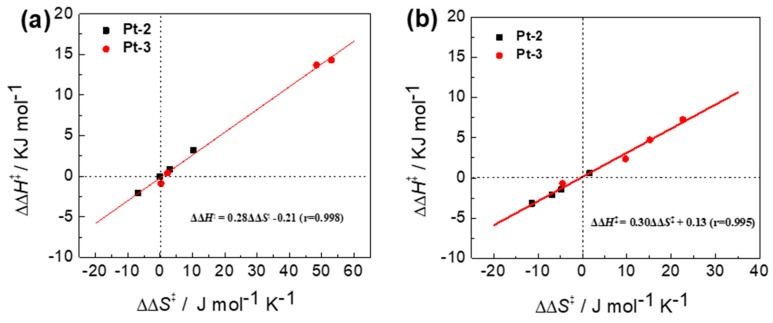
Enthalpy–entropy compensation plot for the differential parameters of the enantiomers of **2** obtained in the photocyclodimerization of AC catalyzed by **Pt-2** (■) and **Pt-3** (**●**). (**a**) of the ee of **2,** (**b**) of the ee of **3**.

All of the ΔΔ*H*^‡^ values obtained for **2** and **3** in this study are plotted against the relevant ΔΔ*S*^‡^ values to give the enthalpy–entropy compensation plot (Figure 4) [29]. The differential parameters obtained at 365 and 532 nm falled on a single straight line, satisfying the equation ΔΔ*H*^‡^ = 0.28ΔΔ*S*^‡^ − 0.21 (r = 0.998), ΔΔ*H*^‡^ = 0.30ΔΔ*S*^‡^ + 0.13 (r = 0.995) for **2** and **3**, respectively. This is reasonable, as the chirality origin of this enantiodifferentiating photodimerization is from the charity of the CD cavity. From the slope of the line, the equipodal temperature for the formation of **2** and **3** are determined to be 280 K (7 °C) and 300 K (27 °C), separately. 

Energy transfer efficiency from the sensitizer to AC at different temperatures was further investigated by the quenching of the phosphorescence of the complexes **Pt-3** with AC as the triplet quencher. The emission of the complex was progressively reduced with the increase of the concentration of AC due to the triplet energy transfer from the complex to AC. It should be pointed out that dynamic and static quenching by the free and bonded AC are responsible for the quenching of phosphorescence of the sensitizer, and static quenching process should be much more efficient and highly dependent on the distance between γ-CD and Schiff-base complexes unit. Stern–Volmer quenching curves were constructed with the quenching data which shows a good linear relationship in the temperature range of 0–30 °C (Figure S11). In accordance with association constants, at the lower temperature **Pt-3** showed much larger *K_SV_*, which demonstrates that static quenching in **Pt-3** is much more efficient due to the intimate contact of AC with Schiff-base Pt complex unit. Whereas, much higher *K_SV_* at lower temperature together with the fact that **Pt-3** showed much higher overall association constant *K_1_K_2_*, jointly confirmed that thermodynamic photoreaction competed with the kinetic ones, so that decreases the enantioselectivity of the photoreaction.

## 3. Materials and Methods 

### 3.1. Chemicals and Instruments

All the chemicals used in synthesis are analytically pure and were used as received. Solvents were dried and distilled before use for synthesis. ^1^H-NMR and ^13^C-NMR spectra were recorded at room temperature on Bruker AMX-400 (operating at 400 MHz for ^1^H-NMR and 151 MHz for ^13^C-NMR) with TMS as the internal standard (Bruker, Bremen, Germany). High resolution mass spectrum (HRMS) data were measured in quadrupole time-of-fight liquid chromatography-mass spectrometer (Q-TOF-LCMS) (Shimadzu, Tokyo, Japan) and matrix-assisted laser desorption ionization time-of-fight mass spectrometer (MALDI-TOF MS, Bruker, Bremen, Germany). UV-vis. spectra were obtained on JASCO v-650 (Jasco, Tokyo, Japan). CD spectra were acquired using J-1500 CD spectrometer (Jasco, Tokyo, Japan). Fluorescence spectra and Fluorescence lifetime decay were taken on Fluoromax-4 spectrofluorometer (Horiba, New Jersey, NJ, USA). Photocyclodimerization data was acquired by high-performance liquid chromatography (HPLC) (UFLC SHIMADZU system equipped with SPD-20A and RF-20A as a detector, Shimadzu, Tokyo, Japan).

### 3.2. Photoreaction and Product Analysis

**Photolyses.** The mixture aqueous solution of AC (0.2 mM) and sensitizer (**Pt-1**, **Pt-2,** and **Pt-3** in different concentration) was irradiated for 2 h under an argon atmosphere using a LED lamp (365 nm) or a diode pumped solid state (DPSS) laser (532 nm). 

**HPLC analysis.** The resulting solution was analyzed by Chiral HPLC, performed on a tandem column of Inertsil ODS-2 and Daicel Chiralcel OJ-R, and operated at 35 °C using 0.1% trifluoroacetic acid (TFA) dissolved in H_2_O and acetonitrile (62:38, volume ratio), at a flow rate of 0.5 mL/min. The relative yield and *ee* value were determined from the peak area of HPLC chromatogram.

### 3.3. Synthesis

**Synthesis of Pt-1**: After degassing of DMSO (5 mL), compound **L1** (180 mg, 0.5 mmol), K_2_PtCl_4_ (210 mg, 0.5 mmol), and K_2_CO_3_ (210 mg, 1.5 mmol) were added, the flask was placed under vacuum and backfilled with argon several times. Then the reaction mixture was heated to 80 °C for 18 h. The solvent was evaporated under reduced pressure at 80 °C and the crude product was washed with water (2 × 100 mL) to give the reddish-brown product (230 mg, 83.18%). ^1^H-NMR (400 MHz, DMSO-*d*_6_) δ (ppm): 9.54 (s, 1H), 9.52 (s, 1H), 8.78 (s, 1H), 8.31 (d, *J* = 8.8 Hz, 1H), 8.04 (d, *J* = 8.1, Hz, 1H), 7.94–7.82 (m, 2H), 7.67–7.43 (m, 2H), 7.10 (d, *J* = 8.3 Hz, 2H), 6.86–6.62 (m, 2H). ^13^C-NMR (101 MHz, DMSO-*d*_6_) δ (ppm): 164.37, 151.17, 144.79, 143.90, 136.07, 135.69, 135.37, 128.72, 122.13, 121.85, 121.22, 121.03, 120.55, 117.64, 116.29, 116.17, 115.21, 113.60, 112.19. HRMS (ESI-): *m*/*z* calcd for C_21_H_14_N_2_O_4_Pt [M − H]^−^, 552.0522, found [M − H]^−^, 552.0519. 

**Synthesis of Pt-2**: The solution of compound **Pt-1** (55.3 mg, 0.1 mmol) in DMF (5 mL) were added **EDC** (1-(3-dimethylaminopropyl)-3-ethylcarbodiimide) (33 μL, 0.15 mmol) and **HOBT** (1-hydroxybenzotriazole) (20.3 mg, 0.15 mmol), and the mixture was stirred at −20 °C for 3 h. Then compound 6^A^-amino-6^A^-deoxy-β-CD (136 mg, 0.12 mmol, 1.2 equiv.) was added, and the mixture was stirred at room temperature for another 6 h. After completion of the reaction, the mixture was dropwise added into acetone (200 mL) to obtain the crude product as a red precipitate, which was purified by reverse phase chromatography, using ethanol aqueous solution in gradient elution from 0–50% to afford the product as red solid (45.6 mg, 27.32%). ^1^H-NMR (400 MHz, DMSO-*d*_6_) δ (ppm): 9.51 (d, *J* = 6.8 Hz, 2H), 8.84 (s, 1H), 8.53–8.39 (m, 2H), 7.86 (d, *J* = 9.1 Hz, 3H), 7.59 (d, *J* = 6.1 Hz, 2H), 7.12 (d, *J* = 8.6 Hz, 2H), 6.81 (d, *J* = 7.0 Hz, 2H), 5.79 (d, *J* = 18.8 Hz, 14H), 5.14–4.68 (m, 7H), 4.49 (t, *J* = 36.1 Hz, 7H), 4.08 (d, *J* = 7.2 Hz, 1H), 3.94–3.80 (m, 1H), 3.63 (d, *J* = 26.2, 40H). ^13^C-NMR (151 MHz, DMSO-*d*_6_) δ (ppm): 165.53, 165.26, 165.09, 152.34, 152.11, 146.86, 144.74, 136.35, 136.29, 136.12, 133.96, 127.18, 122.43, 122.35, 121.82, 121.71, 116.98, 116.63, 116.23, 102.77, 102.41, 102.03, 84.83, 82.10, 81.93, 81.76, 81.70, 73.51, 73.44, 72.89, 72.76, 72.62, 72.56, 72.48, 72.39, 70.35, 60.59, 60.42, 60.34, 60.23, 59.86. MALDI-TOF-HRMS: *m*/*z* calcd for C_63_H_83_N_3_O_37_Pt M, 1668.4353, [M + H]^+^, 1669.4431; found [M + H]^+^, 1669.4962.

**Synthesis of Pt-3**: The synthesis procedure is the same as **Pt-2**. ^1^H-NMR (400 MHz, DMSO-*d*_6_) δ (ppm): 9.50 (s, 2H), 8.81 (s, 1H), 8.44 (d, 1H), 7.86 (d, *J* = 8.0 Hz, 3H), 7.56 (s, 2H), 7.11 (d, *J* = 8.7 Hz, 2H), 6.77 (t, 2H). ^13^C-NMR (151 MHz, DMSO-*d*_6_) δ (ppm): 165.68, 165.20, 165.04, 146.84, 144.64, 136.31, 133.84, 122.35, 121.72, 117.01, 116.61, 116.02, 102.86, 102.18, 101.94, 81.48, 81.04, 80.51, 73.05, 72.80, 72.56, 60.36, 56.43. MALDI-TOF-HRMS: *m*/*z* calcd for C_69_H_93_N_3_O_42_Pt [M], 1830.4881, [M + Na]^+^, 1853.4779, [M + K]^+^, 1869.4518, found M^+^, 1830.4833, [M + Na]^+^, 1853.4727, [M + K]^+^, 1869.4523.

## 4. Conclusions

In conclusion, a new β-CD based sensitizer/host **Pt-2** was synthesized and was applied for visible-light-driven photocatalytic supramolecular enantiodifferentiating dimerization of 2-anthracenecarboxylic acid (AC) through triplet-triplet annihilation. The photoreaction mediated with **Pt-2** failed to form irregular chiral slipped 5, 8: 9′, 10′-cyclodimers due to aggregation of **Pt-2** in water, thus hard to form the 2:2 complex with AC. Decreasing the concentration of sensitizers/hosts significantly enhanced the enantioselectivities of the optical photodimers **2** and **3** when employing **Pt-2**, and **Pt-3** as the hosts as well as the sensitizers, and was triggered by 532 nm laser, making them good candidates for organic photocatalyst. The photolysis of AC through triplet-triplet annihilation sensitized by **Pt-3** displayed significant temperature dependency, the ee value of photodimer **2** was enhanced from 2.1% to 31.6% when increasing the temperature from 0 °C to 30 °C in the presence of 5% equivalent of the photocatalyst. This is unprecedented as for traditional enantiodifferentiating photodimerization of AC, the enantioselectivity was deduced with the increasing temperature. This work presents a new strategy for catalytic supramolecular photochirogenesis, and provides a more comprehensive understanding for photochirogenesis through triplet-triplet annihilation.

## Figures and Tables

**Table 1:** Photocyclodimerization of **AC** (0.2 mM) mediated by **Pt-1** and **Pt-2** *^a^*.

Sen.	λ/nm	[Sen.]/μM	Conv% *^b^*	Relative Yield (%) *^b^*						*ee*% *^b^*			
				1	2	3	4	5	6	2	3	5	6
**β-CD**	365	100	37	42	31	15	9	2	1	−1	0	46	31 [49]
**γ-CD**	365	10	97	40	37	14	9	- *^c^*	- *^c^*	8	4	- *^c^*	- *^c^*
**Pt-1**	532	50	53	37	37	16	10	- *^c^*	- *^c^*	- *^c^*	- *^c^*	- *^c^*	- *^c^*
**Pt-2**	365	1	90	39	36	15	9	- *^c^*	- *^c^*	0.0	0.3	- *^c^*	- *^c^*
		10	93	42	36	14	8	- *^c^*	- *^c^*	1.3	0.3	- *^c^*	- *^c^*
		50	98	39	36	15	10	- *^c^*	- *^c^*	−0.1	0.7	- *^c^*	- *^c^*
		100	97	38	36	16	10	- *^c^*	- *^c^*	−5.1	2.3	- *^c^*	- *^c^*
	532	1	1	40	38	14	9	- *^c^*	- *^c^*	1.6	−0.2	- *^c^*	- *^c^*
		10	75	40	38	14	8	- *^c^*	- *^c^*	2.2	−2.0	- *^c^*	- *^c^*
		50	80	40	39	13	8	- *^c^*	- *^c^*	2.0	−2.8	- *^c^*	- *^c^*
		100	97	41	40	12	7	- *^c^*	- *^c^*	1.8	−3.5	- *^c^*	- *^c^*

*^a^* Irradiation with a light-emitting diode (LED) lamp at 365 nm for 2 h or a solid state laser at 532 nm for 2 h, in borate buffer solution (pH 9.0), 20 °C. *^b^* Conversion ratio, relative yield and *ee* determined by chiral HPLC. *^c^* not detected.

**Table 2:** Photocyclodimerization of AC (0.2 mM) with **Pt-2** as the sensitizer/host under different temperatures *^a^*.

λ/nm	T/°C	Conv% *^b^*	[Sen.]	Relative Yield (%) *^b^*				*ee*% *^b^*		HH/HT	Anti/syn	Anti/syn
			/mM	1	2	3	4	2	3		1/2	3/4
365	0	98	0.01	39	38	14	9	1.6	3.4	0.3	1.0	1.5
	10	96	0.01	41	40	12	7	1.5	3.2	0.2	1.0	1.6
	20	93	0.01	42	37	13	8	1.3	2.9	0.3	1.1	1.7
	30	71	0.01	44	36	12	8	1.2	2.7	0.3	1.2	1.5
	0	80	0.1	37	40	13	10	−7.9	5.9	0.3	0.9	1.4
	10	96	0.1	37	37	15	10	−7.0	3.5	0.3	1.0	1.5
	20	97	0.1	38	36	16	10	−5.1	2.3	0.4	1.0	1.5
	30	96	0.1	37	34	18	11	−0.7	1.2	0.4	1.1	1.6
532	0	53	0.01	43	38	12	7	3.7	−3.2	0.2	1.1	1.8
	10	54	0.01	42	39	12	7	3.4	−2.9	0.2	1.1	1.7
	20	75	0.01	40	38	14	8	2.2	−2.0	0.3	1.1	1.8
	30	76	0.01	42	37	14	8	0.5	−2.1	0.3	1.1	1.9
	0	75	0.1	42	37	12	9	5.0	1.6	0.3	1.1	1.4
	10	89	0.1	41	37	12	9	2.9	−1.1	0.3	1.1	1.4
	20	97	0.1	41	40	12	7	1.8	−3.5	0.2	1.0	1.6
	30	95	0.1	45	40	9	5	0.3	−5.3	0.2	1.1	1.8

*^a^* Irradiation with a LED lamp at 365 nm for 2h or a solid state laser at 532 nm for 2 h, in borate buffer solution (pH 9.0). *^b^* Conversion ratio, relative yield and *ee* determined by chiral high-performance liquid chromatography (HPLC).

**Table 3:** Differential activation parameters determined from the temperature dependence of the *ee* of cyclodimers **2** and **3** in the photocyclodimerization of AC catalyzed by **Pt-2.**

[Sen.]/mM	λ/nm	Dimer 2		Dimer 3	
		ΔΔ*H*^‡^ (KJ mol^−1^)	ΔΔ*S*^‡^ (J mol^−1^ K^−1^)	ΔΔ*H*^‡^ (KJ mol^−1^)	ΔΔ*S*^‡^ (J mol^−1^ K^−1^)
0.01	365	−0.2	−0.4	−0.3	−0.6
	532	−1.5	−4.7	0.6	1.6
0.1	365	3.2	10.3	−2.1	−6.8
	532	−2.1	−6.9	−3.2	−11.4

**Table 4:** Photocyclodimerization of **AC** (0.2 mM) and **Pt-3** mediated by temperature *^a^*.

λ/nm	T/°C	Conv% *^b^*	[sen.] /mM	Relative Yield (%) *^b^*				*ee*% *^b^*		HH/HT	Anti/syn 1/2	Anti/syn 3/4
				1	2	3	4	2	3			
365	0	74	0.01	37	39	13	11	5.5	4.9	0.3	0.9	1.2
	10	90	0.01	36	38	14	11	5.7	9.9	0.3	0.9	1.2
	20	92	0.01	37	38	14	11	6.0	10.7	0.3	1.0	1.3
	30	95	0.01	37	37	15	11	6.3	10.2	0.3	1.0	1.4
	0	85	1	25	24	26	26	21.1	−11.8	1.1	1.1	1.0
	10	88	1	28	28	23	22	21.4	−12.7	0.8	1.0	1.1
	20	81	1	28	29	23	21	20.8	−12.8	0.8	1.0	1.1
	30	87	1	30	30	21	18	19.1	−13.4	0.7	1.0	1.2
532	0	63	0.01	38	54	5	3	2.1	−12.4	0.1	0.7	1.9
	10	81	0.01	37	56	5	2	16.7	−10.5	0.1	0.8	2.3
	20	90	0.01	34	56	7	4	27.4	−4.5	0.1	0.6	1.9
	30	95	0.01	35	59	4	2	31.6	−3.0	0.1	0.6	1.8
	0	93	1	39	44	11	6	−11.9	−22.3	0.2	0.9	1.7
	10	93	1	36	47	11	6	1.3	−20.3	0.2	0.8	2.0
	20	96	1	33	48	13	6	13.0	−12.7	0.2	0.7	2.0
	30	99	1	32	47	14	7	16.9	−7.6	0.3	0.7	2.0

*^a^* Irradiation with a LED lamp at 365 nm for 2 h or a solid state laser at 532 nm for 2 h. In borate buffer solution (pH 9.0). *^b^* Conversion ratio, relative yield and *ee* determined by chiral HPLC.

**Table 5:** Differential activation parameters determined from the temperature dependence of the ee of cyclodimers **2** and **3** in the photocyclodimerization of AC catalyzed by **Pt-3.**

Host	[Sen.]/mM	λ/nm	Dimer 2		Dimer 3	
			ΔΔ*H*^‡^ (KJ mol^−1^)	ΔΔ*S*^‡^ (J mol^−1^ K^−1^)	ΔΔ*H*^‡^ (KJ mol^−1^)	ΔΔ*S*^‡^ (J mol^−1^ K^−1^)
**Pt-3**	0.01	365	0.4	2.3	2.4	9.7
		532	14.3	53.0	4.7	15.2
	1	365	−0.9	0.3	−0.7	−4.5
		532	13.7	48.4	7.3	22.6

## Supplementary Materials

The following are available online: Scheme S1: synthesis of **Pt-1** and **Pt-2**, Figure S1-3: ^1^H-NMR; ^13^C-NMR and HRMS spectra of **Pt-2**, Figure S4: ^1^H-NMR spectra of **Pt-2** in different deuterated solvent, Figure S5: ^1^H-NMR spectra of the mixture of **Pt-2** and AC, Figure S6a: UV-vis spectra of **Pt-1**, **Pt-2**, and **Pt-3**, Figure S6b: Normalized UV-vis spectra of **Pt-2** at different concentrations, Figure S7: Emission spectra of **Pt-2**, Figure S8: Phosphorescence quenching spectra of **Pt-2** upon increasing AC, Figure S9: CD spectra of **Pt-2** and **Pt-3**, Figure S10: Job plots of **Pt-2** and **Pt-3**, Figure S11: Stern-Volmer plots of **Pt-3**; Table S1: photophysical properties of the sensitizers/hosts **Pt-1**, **Pt-2**, and **Pt-3**, and Stern–Volmer quenching constant (*K_SV_*) between sensitizers/host and AC at 20 °C. Table S2: association constants of **Pt-3** and AC at a different temperature.

## References

[1] Huo H., Shen X., Wang C., Zhang L., Rose P., Chen L.A., Harms K., Marsch M., Hilt G., Meggers E. (2014). Asymmetric photoredox transition-metal catalysis activated by visible light. Nature.

[2] Nakamura A., Inoue Y. (2005). Electrostatic manipulation of enantiodifferentiating photocyclodimerization of 2-anthracenecarboxylate within γ-cyclodextrin cavity through chemical modification. inverted product distribution and enhanced enantioselectivity. J. Am. Chem. Soc..

[3] Yang C., Nakamura A., Fukuhara G., Origane Y., Mori T., Wada T., Inoue Y. (2006). Pressure and temperature-controlled enantiodifferentiating [4 + 4] photocyclodimerization of 2-anthracenecarboxylate mediated by secondary face- and skeleton-modified γ-cyclodextrins. J. Org. Chem..

[4] Yang C., Nakamura A., Wada T., Inoue Y. (2006). Enantiodifferentiating photocyclodimerization of 2-anthracenecarboxylic acid mediated by γ-cyclodextrins with a flexible or rigid cap. Org. Lett..

[5] Yang C., Mori T., Inoue Y. (2008). Supramolecular enantiodifferentiating photocyclodimerization of 2-anthracenecarboxylate mediated by capped γ-cyclodextrins: Critical control of enantioselectivity by cap rigidity. J. Org. Chem..

[6] Yang C., Mori T., Origane Y., Ho Ko Y., Selvapalam N., Kim K., Inoue Y. (2008). Highly stereoselective photocyclodimerization of γ-cyclodextrin-appended anthracene mediated by γ-cyclodextrin and cucurbit[8]uril: A dramatic steric effect operating outside the binding site. J. Am. Chem. Soc..

[7] Wang Q., Yang C., Fukuhara G., Mori T., Liu Y., Inoue Y. (2011). Supramolecular FRET photocyclodimerization of anthracenecarboxylate with naphthalene-capped γ-cyclodextrin. Beilstein J. Org. Chem..

[8] Wang Q., Yang C., Ke C., Fukuhara G., Mori T., Liu Y., Inoue Y. (2011). Wavelength-controlled supramolecular photocyclodimerization of anthracenecarboxylate mediated by γ-cyclodextrins. Chem. Commun..

[9] Yang C., Ke C., Liang W., Fukuhara G., Mori T., Liu Y., Inoue Y. (2011). Dual supramolecular photochirogenesis: Ultimate stereocontrol of photocyclodimerization by a chiral scaffold and confining host. J. Am. Chem. Soc..

[10] Yao J., Yan Z., Ji J., Wu W., Yang C., Nishijima M., Fukuhara G., Mori T., Inoue Y. (2014). Ammonia-driven chirality inversion and enhancement in enantiodifferentiating photocyclodimerization of 2-anthracenecarboxylate mediated by diguanidino-γ-cyclodextrin. J. Am. Chem. Soc..

[11] Yi J., Liang W., Wei X., Yao J., Yan Z., Su D., Zhong Z., Gao G., Wu W., Yang C. (2018). Switched enantioselectivity by solvent components and temperature in photocyclodimerization of 2-anthracenecarboxylate with 6 A,6 X-diguanidio-γ-cyclodextrins. Chin. Chem. Lett..

[12] Yang C., Ke C., Kahee F., Yuan D., Mori T., Inoue Y. (2008). pH-Controlled supramolecular enantiodifferentiating photocyclodimerization of 2-anthracenecarboxylate with capped γ-cyclodextrins. Aust. J. Chem..

[13] Yang C., Wang Q., Yamauchi M., Yao J., Zhou D., Nishijima M., Fukuhara G., Mori T., Liu Y., Inoue Y. (2014). Manipulating γ-cyclodextrin-mediated photocyclodimerization of anthracenecarboxylate by wavelength, temperature, solvent and host. Photochem. Photobiol. Sci..

[14] Huang Q., Jiang L., Liang W., Gui J., Xu D., Wu W., Nakai Y., Nishijima M., Fukuhara G., Mori T. (2016). Inherently chiral azonia[6]helicene-modified β-cyclodextrin: Synthesis, characterization, and chirality sensing of underivatized amino acids in water. J. Org. Chem..

[15] Liu R., Zhang Y., Liang W., Wu W., Huang Q., Yu X., Xu W., Zhou D., Yang C. (2018). Temperature-driven braking of γ-cyclodextrin-curcubit[6]uril-cowheeled[4]rotaxanes. Chin. Chem. Lett..

[16] Kawanami Y., Umehara H., Mizoguchi J., Nishijima M., Fukuhara G., Yang C., Mori T., Inoue Y. (2013). Cross- versus homo-photocyclodimerization of anthracene and 2-anthracenecarboxylic acid mediated by a chiral hydrogen-bonding template. Factors controlling the cross-/homo-selectivity and enantioselectivity. J. Org. Chem..

[17] Kawanami Y., Pace T.C., Mizoguchi J., Yanagi T., Nishijima M., Mori T., Wada T., Bohne C., Inoue Y. (2009). Supramolecular complexation and enantiodifferentiating photocyclodimerization of 2-anthracenecarboxylic acid with 4-aminoprolinol derivatives as chiral hydrogen-bonding templates. J. Org. Chem..

[18] Kawanami Y., Katsumata S.Y., Nishijima M., Fukuhara G., Asano K., Suzuki T., Yang C., Nakamura A., Mori T., Inoue Y. (2016). Supramolecular photochirogenesis with a higher-order complex: Highly accelerated exclusively head-to-head photocyclodimerization of 2-anthracenecarboxylic acid via 2:2 complexation with prolinol. J. Am. Chem. Soc..

[19] Bauer A., Westkamper F., Grimme S., Bach T. (2005). Catalytic enantioselective reactions driven by photoinduced electron transfer. Nature.

[20] Gui J., Yan Z., Peng Y., Yi J., Zhou D., Su D., Zhong Z., Gao G., Wu W., Yang C. (2016). Enhanced head-to-head photodimers in the photocyclodimerization of anthracenecarboxylic acid with a cationic pillar[6]arene. Chin. Chem. Lett..

[21] Alagesan M., Kanagaraj K., Wan S., Sun H., Su D., Zhong Z., Zhou D., Wu W., Gao G., Zhang H. (2016). Enantiodifferentiating [4 + 4] photocyclodimerization of 2-anthracenecarboxylate mediated by a self-assembled iron tetrahedral coordination cage. J. Photochem. Photobiol. A: Chem..

[22] Wada T., Nishijima M., Fujisawa T., Sugahara N., Mori T., Nakamura A., Inoue Y. (2003). Bovine serum albumin-mediated enantiodifferentiating photocyclodimerization of 2-anthracenecarboxylate. J. Am. Chem. Soc..

[23] Nishijima M., Pace T.C., Nakamura A., Mori T., Wada T., Bohne C., Inoue Y. (2007). Supramolecular photochirogenesis with biomolecules. Mechanistic studies on the enantiodifferentiation for the photocyclodimerization of 2-anthracenecarboxylate mediated by bovine serum albumin. J. Am. Chem. Soc..

[24] Yang C., Inoue Y. (2014). Supramolecular photochirogenesis. Chem. Soc. Rev..

[25] Richard B., Dominik L., Maturi M.M., Thorsten B. (2015). Enantioselective catalysis of photochemical reactions. Angew. Chem. Int. Ed..

[26] Yan Z., Wu W., Yang C., Inoue Y. (2015). Catalytic supramolecular photochirogenesis. Supramol. Catal..

[27] Ke C., Yang C., Mori T., Wada T., Liu Y., Inoue Y. (2009). Catalytic enantiodifferentiating photocyclodimerization of 2-anthracenecarboxylic acid mediated by a non-sensitizing chiral metallosupramolecular host. Angew. Chem. Int. Ed..

[28] Brimioulle R., Bach T. (2013). Enantioselective Lewis acid catalysis of intramolecular enone [2+2] photocycloaddition reactions. Science.

[29] Rao M., Kanagaraj K., Fan C., Ji J., Xiao C., Wei X., Wu W., Yang C. (2018). Photocatalytic supramolecular enantiodifferentiating dimerization of 2-anthracenecarboxylic acid through triplet-triplet annihilation. Org. Lett..

[30] Zhao J., Ji S., Guo H. (2011). Triplet–triplet annihilation based upconversion: From triplet sensitizers and triplet acceptors to upconversion quantum yields. RSC Adv..

[31] Singhrachford N.T., Castellano F.N. (2010). Photon upconversion based on sensitized triplet–triplet annihilation. Coordin. Chem. Rev. 3. Photochem. Photobid. A: Chem..

[32] Islangulov R.R., Lott R., Weder R., Castellano F.N. (2007). Noncoherent low-power upconversion in solid polymer films. J. Am. Chem. Soc..

[33] Trupke T., Shalav A., Richards B.S., Würfel P., Green M.A. (2006). Efficiency enhancement of solar cells by luminescent up-conversion of sunlight. Sol. Ene. Mater. Solar Cells.

[34] Kim J.H., Kim J.H. (2012). Encapsulated triplet-triplet annihilation-based upconversion in the aqueous phase for sub-band-gap semiconductor photocatalysis. J. Am. Chem. Soc..

[35] Mattiello S., Monguzzi A., Pedrini J., Sassi M., Villa C., Torrente Y., Marotta R., Meinardi F., Beverina L. (2016). Self-assembled dual dye-doped nanosized micelles for high-contrast up-conversion bioimaging. Adv. Func. Mater..

[36] Monguzzi A., Tubino R., Meinardi F. (2008). Upconversion-induced delayed fluorescence in multicomponent organic systems: Role of Dexter energy transfer. Physical Review B.

[37] Fan C., Wu W., Chruma J.J., Zhao J., Yang C. (2016). Enhanced triplet-triplet energy transfer and upconversion fluorescence through host-guest complexation. J. Am. Chem. Soc..

[38] Griesbeck A., Meierhenrich U.J. (2002). Asymmetric photochemistry and photochirogenesis. Angew. Chem. Int. Ed..

[39] Inoue Y., Ramamurthy V. (2004). Chiral Photochemistry.

[40] Ramamurthy V., Inoue Y. (2011). Supramolecular Photochemistry.

[41] Vallavoju N., Sivaguru J. (2014). Supramolecular photocatalysis: Combining confinement and non-covalent interactions to control light initiated reactions. Chem. Soc. Rev..

[42] Wakai A., Fukasawa H., Yang C., Mori T., Inoue Y. (2012). Theoretical and experimental investigations of circular dichroism and absolute configuration determination of chiral anthracene photodimers. J. Am. Chem. Soc..

[43] Nakamura A., Inoue Y. (2003). Supramolecular catalysis of the enantiodifferentiating [4 + 4] photocyclodimerization of 2-anthracenecarboxylate by γ-cyclodextrin. J. Am. Chem. Soc..

[44] Wei X., Yu X., Zhang Y., Wenting L., Ji J., Yao J., Rao M., Wu W., Yang C. (2019). Enhanced irregular photodimers and switched enantioselectivity by solvent and temperature in the photocyclodimerization of 2-anthracenecarboxylate with modified β-cyclodextrins. J. Photochem. Photobio. A: Chem..

[45] Wei X., Wu W., Matsushita R., Yan Z., Zhou D., Chruma J.J., Nishijima M., Fukuhara G., Mori T., Inoue Y. (2018). Supramolecular photochirogenesis driven by higher-order complexation: Enantiodifferentiating photocyclodimerization of 2-anthracenecarboxylate to slipped cyclodimers via a 2:2 complex with β-cyclodextrin. J. Am. Chem. Soc..

[46] Li Z.Y., Chen J.W., Liu Y., Xia W., Wang L. (2011). The use of calixarenes in asymmetric catalysis. Curr. Org. Chem..

[47] Xu W., Liang W., Wu W., Fan C., Rao M., Su D., Zhong Z., Yang C. (2018). Supramolecular Assembly-Improved Triplet-Triplet Annihilation Upconversion in Aqueous Solution. Chem. Eur. J..

[48] Tamaki T., Kokubu T. (1984). Acceleration of the photodimerization of water-soluble anthracenes included by β- and γ-cyclodextrins. J. Incl. Phenom..

[49] Wu W., Sun J., Ji S., Wu W., Zhao J., Guo H. (2011). Tuning the emissive triplet excited states of platinum(II) Schiff base complexes with pyrene, and application for luminescent oxygen sensing and triplet-triplet-annihilation based upconversions. Dalton Trans.

[50] Kajtár M., Horváth-Toró C., Kuthi E., Szejtli J.A. (1982). A simple rule for predicting circular-dichroism induced in aromatic guests by cyclodextrin hosts in inclusion complexes. Chim.Acad. Sci. Hung.

