# Supplementary material for: Effects of Temperature and Host Concentration on the Supramolecular Enantiodifferentiating [4 + 4] Photodimerization of 2-Anthracenecarboxylate through Triplet-Triplet Annihilation Catalyzed by Pt-Modified Cyclodextrins
Source: Molecules. 2019 Apr 17;24(8):1502. doi: 10.3390/molecules24081502 (PMC6514921; doi:10.3390/molecules24081502)
Supplement: Supplementary file 1 [file molecules-24-01502-s001.pdf]

Supporting Information

for

Effects of Temperature and Host Concentration on the Supramolecular Enantiodifferentiating [4+4] Photodimerization of 2-Anthracenecarboxylate through Triplet-Triplet Annihilation Catalyzed by Pt-Modified Cyclodextrins

Ming Rao, Guo Cheng, Wanhua Wu* and Cheng Yang*

Key Laboratory of Green Chemistry & Technology, College of Chemistry, and Healthy Food Evaluation Research Center, Sichuan University, 29 Wangjiang Road, Chengdu, 610064, China

Contents

1. Synthetic route……………………………………………………………..…S2
2. NMR and HRMS spectra of **Pt-2** and AC………………...……………………....S3
3. Absorption and emission spectra of **Pt-1, Pt-2** and **Pt-3**………………………….S6
4. Photophysical properties of the sensetizers/hosts **Pt-1**, **Pt-2** and **Pt-3**…………..…...S7
5. Binding constants of **Pt-3** and AC at different temperature……………………….S8
6. CD spectra of **Pt-2** and **Pt-3**………………………………………………....…S8
7. Job plots of **Pt-2** and **Pt-3**…………………………………………………...S9
8. Stern-Volmer plots of **Pt-3**……………………………………………………..S9

1. Synthetic route

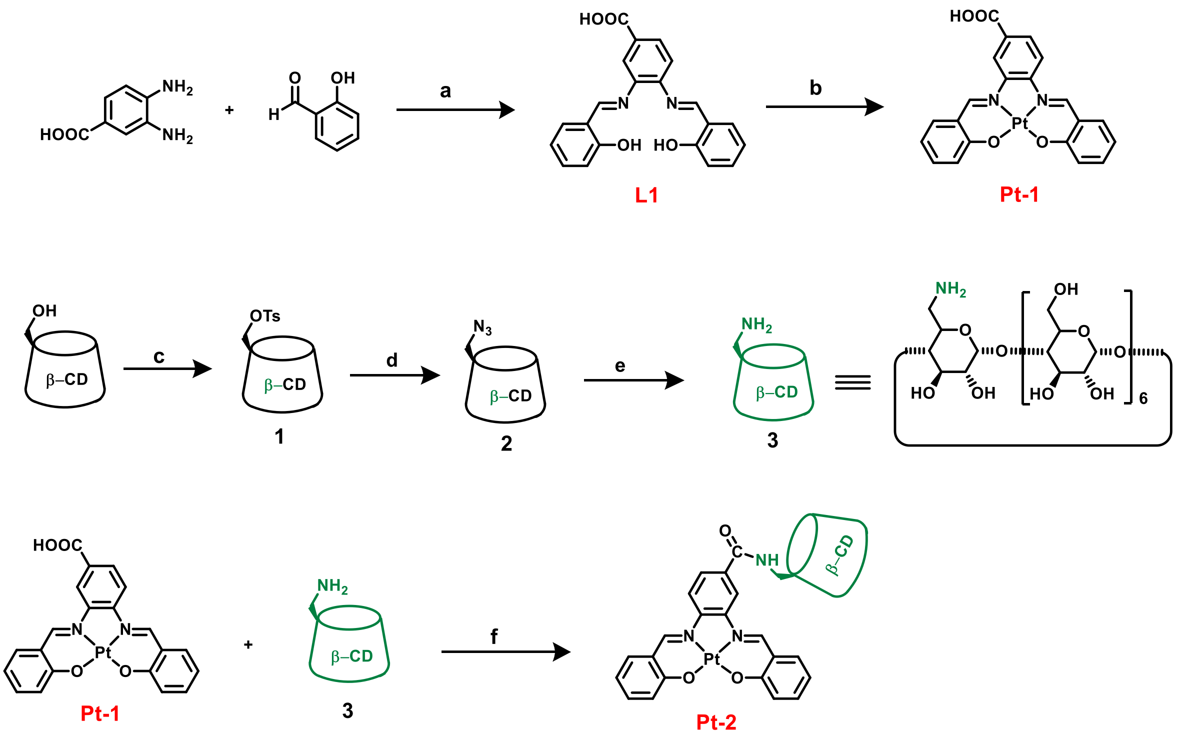

Scheme S1. Synthesis of **Pt-1**, **Pt-2**: a) EtOH, RT, 2 h; b) K_2PtCl_4, K_2CO_3, DMSO, 80 ^oC; c) Pyridine, Paratoluensulfonyl Chloride, RT, 6 h; d) NaN_3, DMF/H_2O (10/1, v/v), 80 ^oC; e) 1) PPh_3, DMF, RT, 6 h; 2) NH_3•H_2O, RT, 1 h; f) 1) EDC, HOBT, -20 ^oC, 2 h; 2) **3**, RT, 6 h;

2. NMR and HRMS spectra of Pt-2 and AC

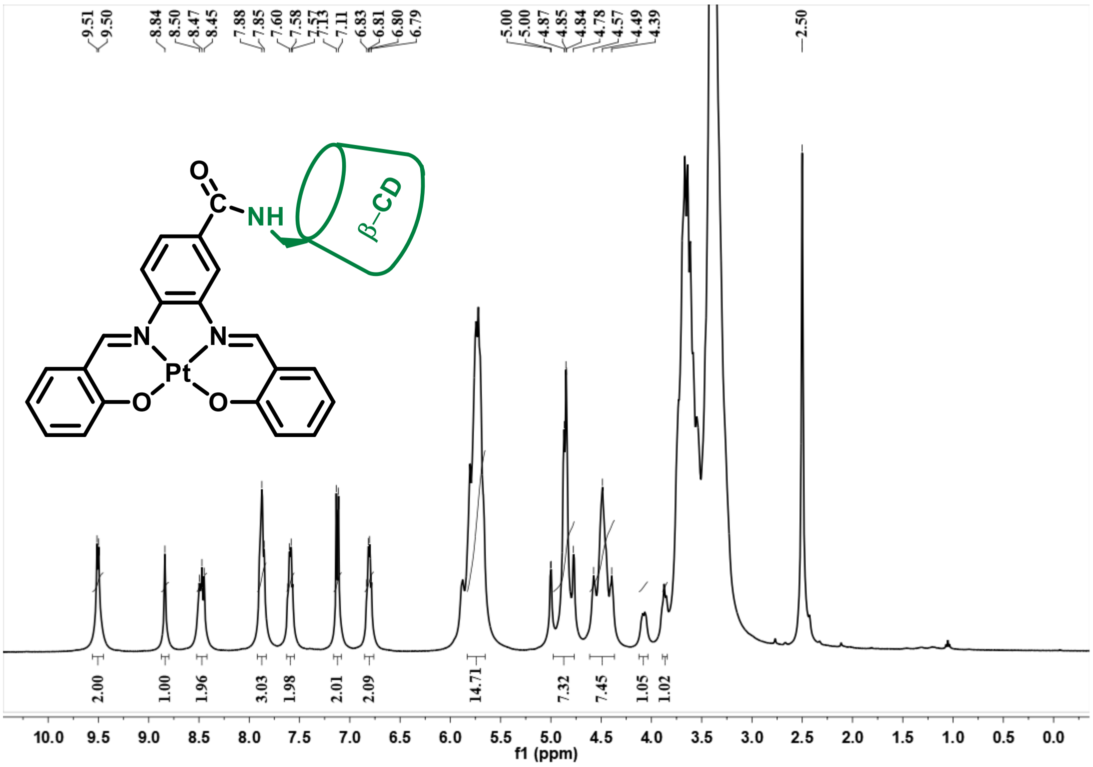

Figure S1. ^{1}H NMR spectrum (400 MHz, DMSO-d_6, room temperature) of **Pt-2.**

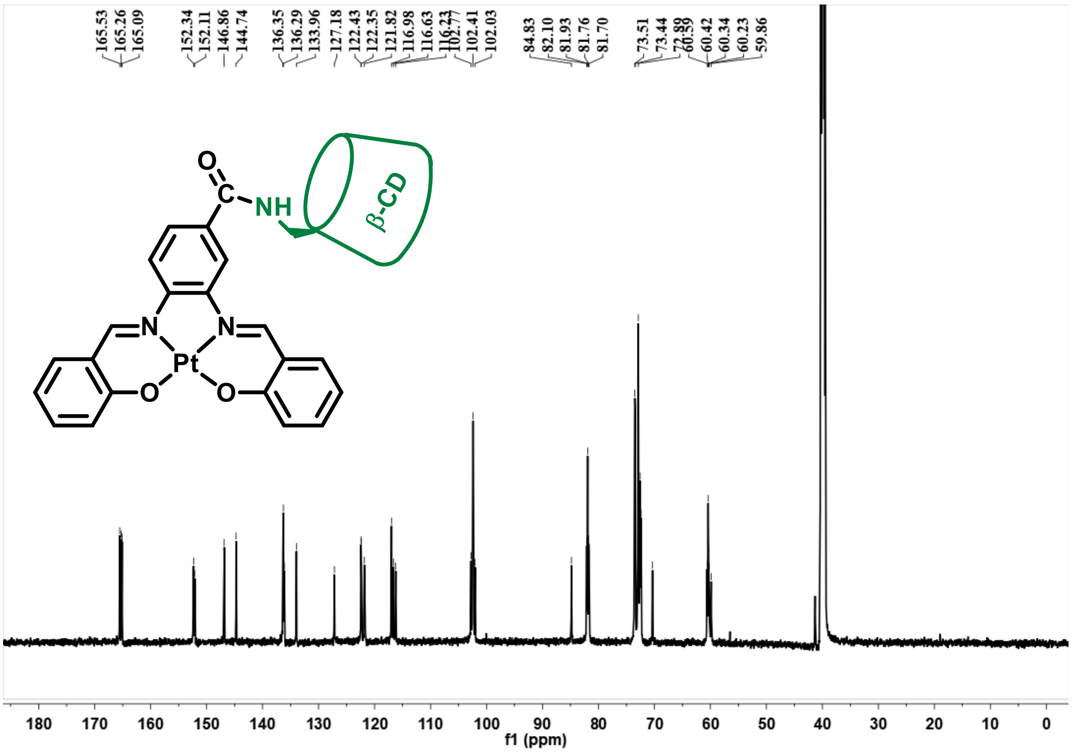

Figure S2. ^{13}C NMR spectrum (151 MHz, DMSO-d_6, room temperature) of **Pt-2.**

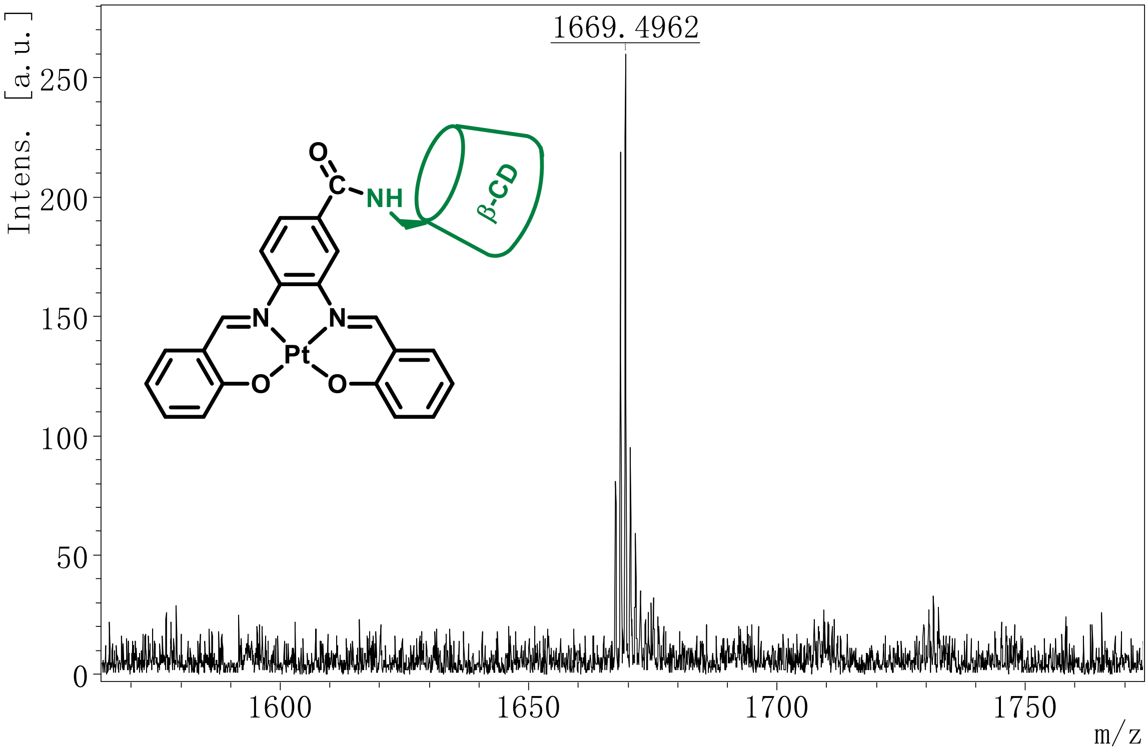

Figure S3. MALDI-TOF-MS of **Pt-2.**

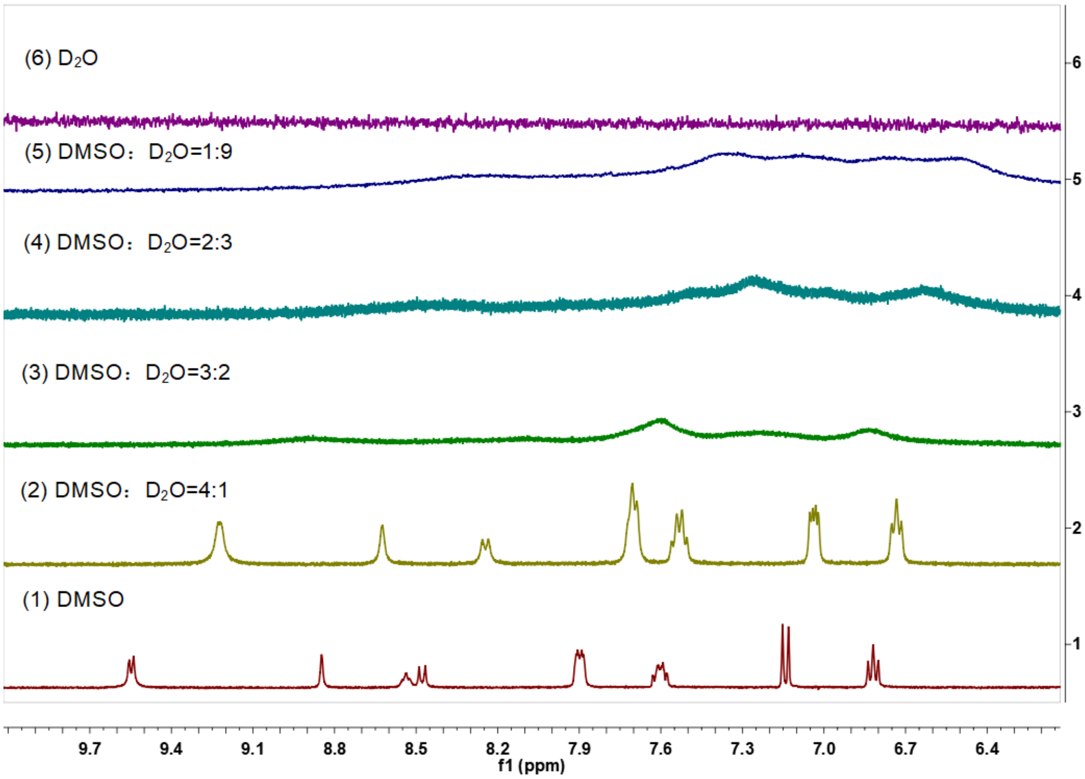

Figure S4. ^{1}H NMR spectra (400 MHz, room temperature) of **Pt-2**. (1) DMSO-d_6, (2) DMSO-d_6 : D_2O=1:4 (v:v), (3) DMSO-d_6 : D_2O=2:3 (v:v) , (4) DMSO-d_6 : D_2O=3:2 (v:v), (5) DMSO-d_6 : D_2O=1:9 (v:v), (6) D_2O.

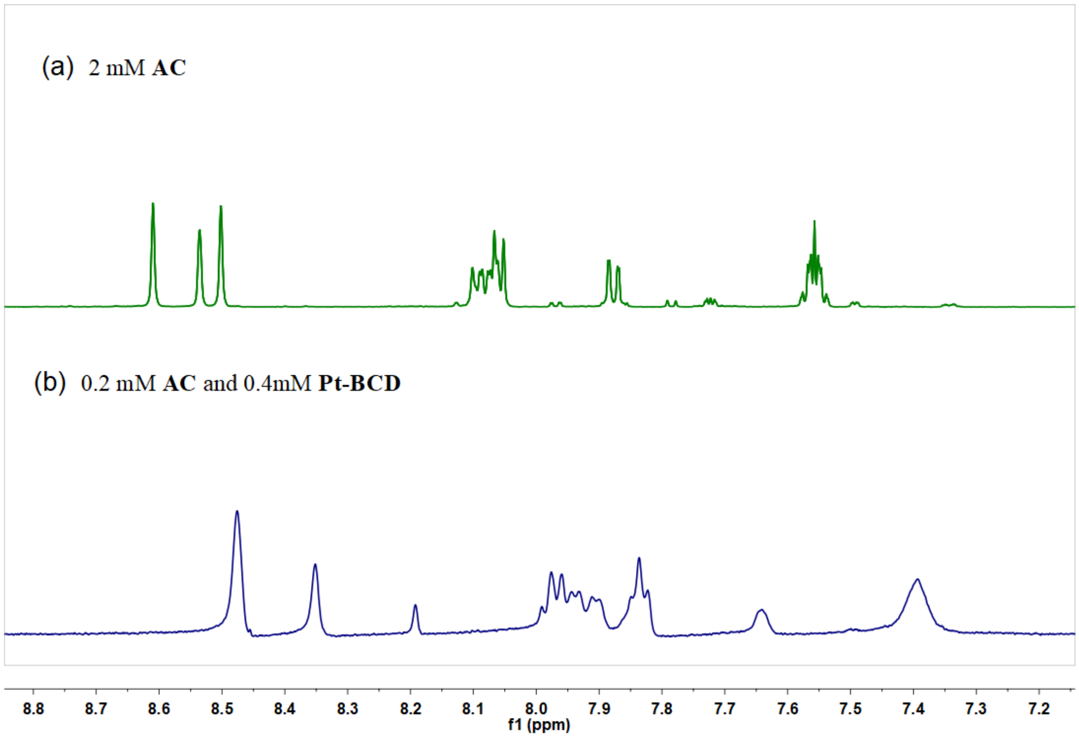

Figure S5. ^{1}H NMR spectra (600 MHz, room temperature) of (a) 2 mM **AC,** (b) 0.2 mM **AC** and 0.4mM **Pt-BCD**, measured in D_2O.

3. Absorption and emission spectra of Pt-1, Pt-2 and Pt-3

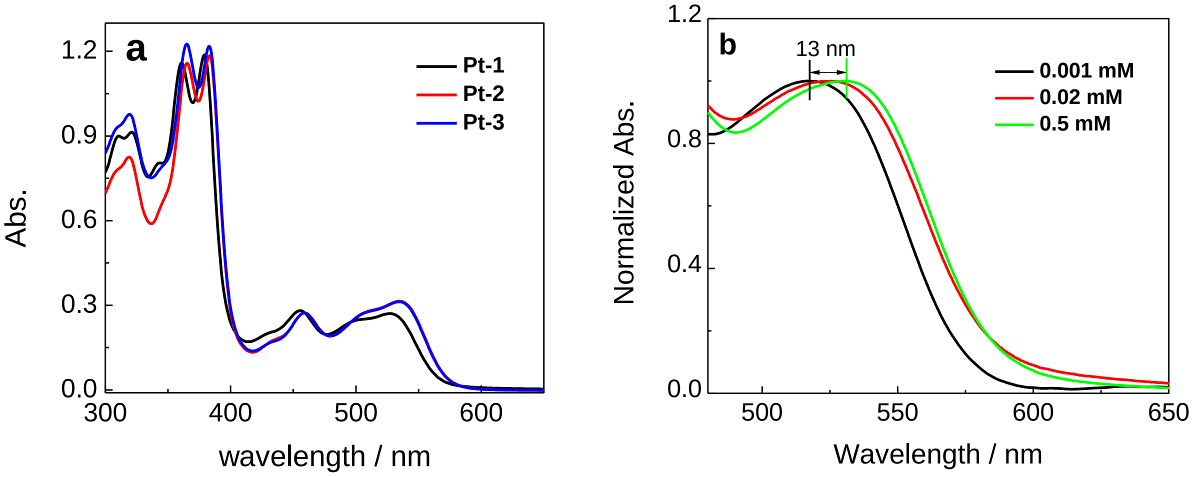

Figure S6. (a) UV-vis absorption spectra of **Pt-1, Pt-2,** and **Pt-3** in DMF solution, $c = 5 \times 10^{-5}$ M, 20 °C. (b) Normalized UV-vis absorption spectra of **Pt-2** at different concentrations in aqueous solution, 20 °C.

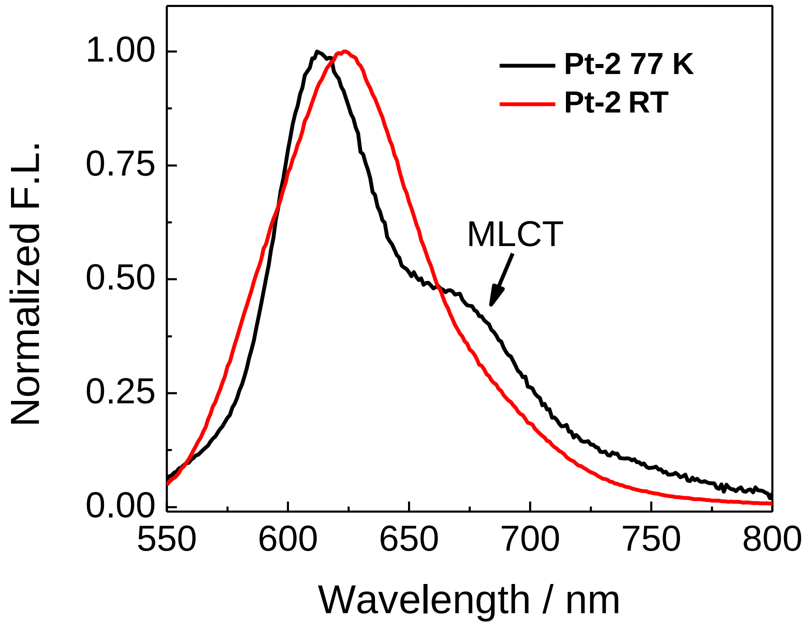

Figure S7. Emission spectra of **Pt-2** measured in ethanol-methanol (4:1, v/v) glass at 77 K (black) and in aqueous solution at room temperature (red) (298 K), λ_{ex} = 532 nm.

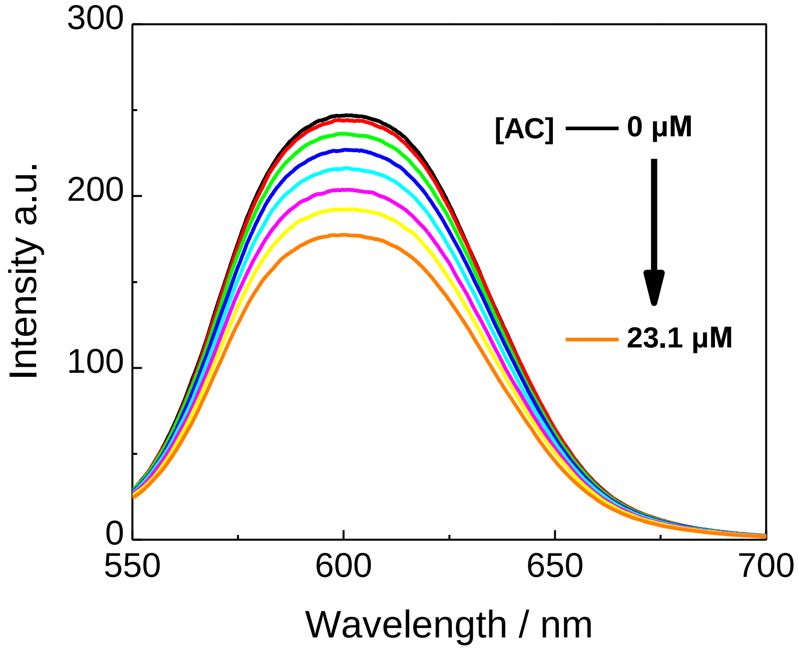

Figure S8. Phosphorescence spectral change of 5 × 10^{-5} M **Pt-2** upon increasing the concentration of AC (0 – 23.1 x 10^{-6} M), λ_{ex} = 532 nm, T = 20 °C.

4. Photophysical properties of the sensetizers/hosts Pt-1, Pt-2, and Pt-3

Table S1. Photophysical properties of the sensetizers/hosts **Pt-1, Pt-2,** and **Pt-3**, and Stern-Volmer quenching constant (K_{SV}) between sensitizers/host and **AC** at 20 °C.

	Abs[a]/nm	ε^b	Em[c]/nm	Φ_F^d	τ_P^e/μs	K_{sv}/10^4 M	K_q /10^9 $M^{-1}s^{-1}$
**Pt-1**	529	5396	599	0.13	2.0	1.28	6.46
**Pt-2**	533	6286	621	0.19	3.1	1.57	5.06
**Pt-3**	533	6267	611	0.20	4.1	2.29	5.65

[a] In DMF at 5 × 10^{-5} M. [b] Molar absorption coefficient at the absorption maxima. [c] Emission in water at RT. [d] Fluorescence quantum yield, rose bengal as standard (Φ_F = 0.11 in EtOH). [e] Phosphorescence lifetimes in deaerated water, 20°C.

5. Binding constants of Pt-3 and AC at different temperature

Table S2. Association Constants for the 1:1 and 1:2 Inclusion Complexes of **Pt-3** with AC Obtained by ITC at Various Temperature.

**T/°C**	**Association constants [a] / M^{-1}**		**K_2 / K_1**	**K_1K_2 (10^6 M^{-2})**
	**K_1[b]**	**K_2[c]**		
0	3810 (68)	5350 (88)	1.4	20.38 (0.006)
25	10400 (160)	719 (25)	0.07	7.48 (0.004)

[a] The values in parentheses the errors. [b] Association constant for **Pt-3** and **AC** making 1:1 complex. [C] Association constant for 1:1 complex and **AC** making 1:2 complex

6. CD spectra of Pt-2 and Pt-3

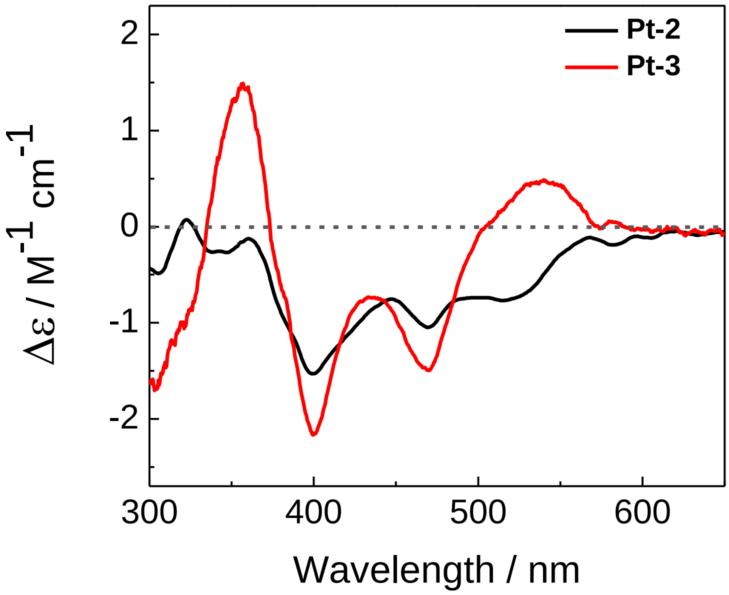

Figure S9. CD Spectra of **Pt-2, Pt-3** in aqueous solution, T=20 °C.

7. Job Plots of Pt-2 and Pt-3

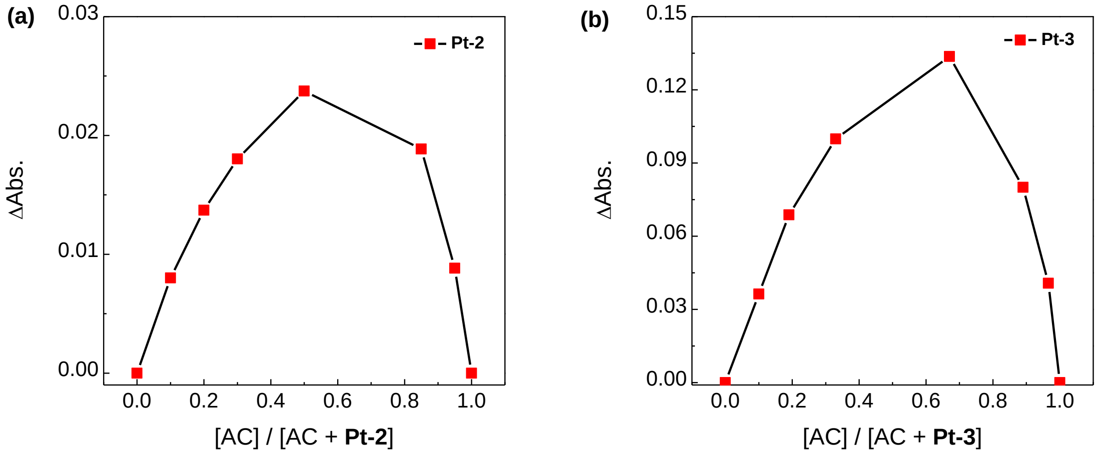

Figure S10. Job plots of the absorption changes at 532 nm of (a) **Pt-2**, (b) **Pt-3** measured by holding the total concentration of [AC]+[sensitizer]=0.2 mM

8. Stern-Volmer plots of Pt-3

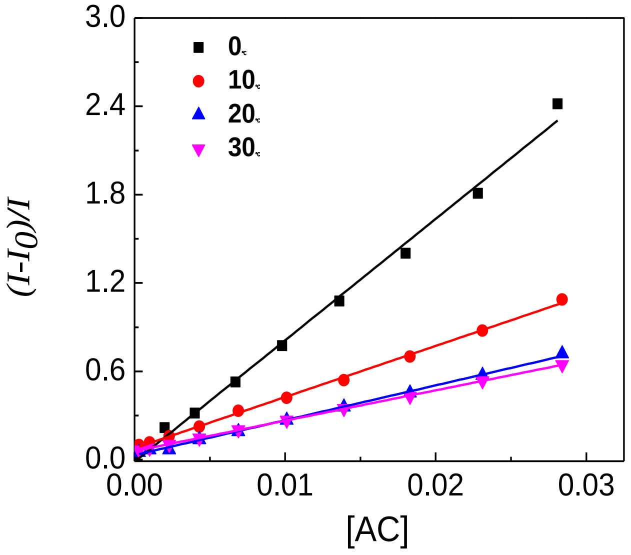

Figure S11. Stern-Volmer plots of **Pt-3** of $(I\text{-}I_0)/I$ versus [AC] in dearated water, $C_{[Pt\text{-}3]}=5 \times 10^{-5}$ M,$C_{[AC]} = 0$ - 2.8×10^{-5} M, T=0, 10, 20, 30 °C.
